# Reviewer Acknowledgements

**DOI:** 10.13023/jah.0603.09

**Published:** 2024-10-01

**Authors:** Randy Wykoff, Emily Wilson

**Affiliations:** East Tennessee State University; East Tennessee State University

**Keywords:** Appalachia, public health

## Abstract

We at the *Journal of Appalachian Health* would like to thank the multitude of reviewers who have volunteered their time, talents, and attention to the journal. Reviewers help us consider the rigor and quality of the submissions we receive, and their willingness to read material ahead of publication ensures we can bring timely research to our readers in Appalachia and further afield.

Every year we reflect on the generosity of a group of people who have volunteered their time, talents, and attention to the journal: our peer reviewers. Reviewers help us consider the rigor and quality of the submissions we receive, and their willingness to read material ahead of publication—and always alongside a raft of competing personal and professional commitments—ensures we can bring timely research to our readers in Appalachia and further afield. As a completely free, open-access publication, the *Journal of Appalachian Health* simply could not remain the public research outlet it is without the generosity of those listed below. In an environment in which all journals increasingly struggle to secure content experts to review submissions, we are especially thankful for the passion and kindness of those who choose to serve our small, but mighty journal.

Alongside our reviewers, we thank the members of our Editorial & Advisory Board, whose institutional affiliations span Appalachia. Additionally, much gratitude is owed to East Tennessee State University (our managing institution), the University of Kentucky (our publisher), and the Appalachian Regional Commission, who for the first time this year provided grant funding to keep our mission afloat. We are excited to see what the coming year will bring as more people join this community in service of all those—near and far—who are proud to call Appalachia home.

Casey P. Balio

Elizabeth A. Beverly

Connie L. Bish

David Blackley

Ty Borders

Billy Brooks

Amy Caruso Brown

Reamer L. Bushardt

Kate Cartwright

Paula Chatterjee

Kaila Christini

Allie Jo Chroust

Stephen Congly

Cory E. Cronin

Elizabeth L. Crouch

Timothy Dasinger

Katie Davis

Kimberly A. Dixon

David L. Driscoll

Paul Erwin

David Eton

Tracy Fasolino

Bradley Firchow

James Fisher

Elliott S. Fisher

Sean J. Fox

Ronald Franks

Craig Gundersen

Angela Hagaman

Rebecca Hagedorn-Hatfield

Gwen Halaas

Sophia Harris

Adam Hege

Michael Hendryx

David Holben

Matthew F Hudson

Danny R. Hughes

Umedjon Ibragimov

Minjae Kim

Thomas Kincer

Brian King

Daniel T. Lackland

Michelle L. Lee

Eugene Lengerich

Mildred Maisonet

Hadii M. Mamudu

Paula Masters

Samuel C. Matheny

Tara L. Maudrie

Bryan McCullick

Renee McGinnis

Julie Miller-Cribbs

Ranjita Misra

Nagabhishek Moka

Justin B. Moore

Donna Petersen

Samuel Pettyjohn

Beth Prusaczyk

Kristin Pullyblank

Miriam C. Purnell

Rebecca Reece

Patricia Nola Eugene Roberson

Rebecca Swinburne Romine

Claire Sadeghzadeh

Chris Salyers

A. Brianna Sheppard

Deborah L. Slawson

Antoinette L. Spector

Ashley Sundin

William Swann

Yuni Tang

Jessica R. Thompson

Martha Tillson

Tracy Trevorrow

Robin C. Vanderpool

Joe Wasserman

Kathy H. Wibberly

